# Positive Allosteric Modulators of Type 5 Metabotropic Glutamate Receptors (mGluR5) and Their Therapeutic Potential for the Treatment of CNS Disorders

**DOI:** 10.3390/molecules16032097

**Published:** 2011-03-02

**Authors:** Richard M. Cleva, M. Foster Olive

**Affiliations:** Department of Psychology, Arizona State University, Tempe, Arizona 85287, USA

**Keywords:** glutamate, receptor, metabotropic, positive allosteric modulator, schizophrenia, addiction, learning, memory, synaptic plasticity

## Abstract

Studies utilizing selective pharmacological antagonists or targeted gene deletion have demonstrated that type 5 metabotropic glutamate receptors (mGluR5) are critical mediators and potential therapeutic targets for the treatment of numerous disorders of the central nervous system (CNS), including depression, anxiety, drug addiction, chronic pain, Fragile X syndrome, Parkinson’s disease, and gastroesophageal reflux disease. However, in recent years, the development of positive allosteric modulators (PAMs) of the mGluR5 receptor have revealed that allosteric activation of this receptor may also be of potential therapeutic benefit for the treatment of other CNS disorders, including schizophrenia, cognitive deficits associated with chronic drug use, and deficits in extinction learning. Here we summarize the discovery and characterization of various mGluR5 PAMs, with an emphasis on those that are systemically active. We will also review animal studies showing that these molecules have potential efficacy as novel antipsychotic agents. Finally, we will summarize findings that suggest that mGluR5 PAMs have pro-cognitive effects such as the ability to enhance synaptic plasticity, improve performance in various learning and memory tasks, including extinction of drug-seeking behavior, and reverse cognitive deficits produced by chronic drug use.

## 1. Glutamateric Neurotransmission

Glutamate is the most prevalent excitatory neurotransmitter within the central nervous system (CNS) and, upon its release into the synaptic cleft, can bind to one of three different ligand-gated ionotropic glutamate receptors (iGluRs): the *N*-methyl-D-aspartate (NMDA) receptor, the α-amino-3-hydroxy-5-methylisoxazole-4-propionic acid (AMPA) receptor, and the kainic acid (KA) receptor. In addition to activation of iGluRs which mediate fast excitatory neurotransmission, glutamate can also bind to G-protein coupled metabotropic glutamate receptors (mGluRs) which mediate slower modulatory neurotransmission.

There are currently eight characterized mGluR subtypes that are subdivided into three distinct groups based upon their neuroanatomical distribution, pharmacological profile, sequence homology, and coupling to intracellular signal transduction cascades [1,2,3,4,5]. Group I mGluRs (mGluR1 and mGluR5) are coupled to G_q/11_ G-proteins and are primarily localized to post synaptic elements in the brain, such as the perisynaptic annulus of dendritic spines. mGluR5 receptors have a broad distribution within the CNS, with moderate to high expression levels in the cerebral cortex, dorsal and ventral striatum, olfactory bulb and tubercle, septal area, hippocampus, inferior colliculus, and spinal nucleus of the trigeminal nerve [6,7,8]. Activation of Group I mGluRs results in increased calcium release from intracellular stores resulting in cell depolarization, enhanced cell excitability, and activation of numerous intracellular signaling molecules such as protein kinase A (PKA), protein kinase C (PKC), mitogen-activated protein kinase (MAPK), extracellular signal-related kinase (ERK), and cAMP response element binding protein (CREB) [3,4,5]. Group II (mGluR2 and mGluR3) and Group III (mGluR4, mGluR6, mGluR7, and mGluR8) mGluRs are G_i/o_-coupled receptors that are often localized on presynaptic terminals. Upon activation, these receptors inhibit the activity of adenylyl cyclase, resulting in a decreased formation of intracellular cyclic adenosine monophosphate (cAMP). These presynaptic mGluRs can act as releasing-regulating autoreceptors that provide negative feedback to inhibit glutamate release. It should be noted that several mGluR subtypes, particularly mGluR3 and mGluR5, have also been identified on glial cells such as astrocytes [9,10,11]. 

**2. mGluR5-NMDA Receptor Interactions**

mGluR5 receptors are physically coupled to NMDA receptors by various scaffolding proteins including PSD-95, Shank, and Homer, as well as via a direct interaction [12]. In addition, mGluR5 receptors are biochemically coupled to NMDA receptor function via PKC [5]. As a result of these molecular and biochemical interactions, activation of mGluR5 receptors results in enhanced functionality of the NMDA receptor [13,14,15,16,17,18]. This mGluR5-NMDA interaction has been observed in numerous brain preparations, whereby activation of mGluR5 receptors with an orthosteric agonist [such as chlorohydroxyphenylglycine (CHPG) or dihydroxyphenylglycine (DHPG)] or a positive allosteric modulator (PAM, see below) potentiates NMDA receptor-mediated responses to exogenously applied glutamate or NMDA. As will be discussed below, this indirect enhancement of NMDA activity by mGluR5 receptor activation has become a recent focus for the development of non-monoaminergic treatments for schizophrenia [5,19,20,21,22,23,24]. In addition, it appears that indirect enhancement of NMDA receptor function by allosteric potentiation of mGluR5 receptors also enhances synaptic plasticity [18,25], performance on certain learning and memory tasks [25,26,27,28], and reverses cognitive and motivational deficits produced by drugs of abuse or NMDA antagonists [29,30,31].

## 3. Discovery and Chemical Properties of mGluR5 Receptor Positive Allosteric Modulators (PAMs)

Positive allosteric modulators (PAMs) of mGluR5 receptor function were originally developed with the intent of indirectly increasing NMDA receptor function toalleviate some of the cognitive deficits associated with schizophrenia, as there is a wealth of evidence suggesting that NMDA hypofunction contributes to cognitive deficits observed in this disorder [19,20,21,32,33,34]. mGluR5 PAMs were hypothesized to be advantageous over orthosteric mGluR5 agonists such as CHPG because the latter compounds: (1) offer poor discrimination between mGluR receptor subtypes due to the high degree of sequence homology of the glutamate binding site; (2) exhibit poor brain penetrance following systemic administration, and (3) cause rapid mGluR5 receptor desensitization. In an effort to circumvent these issues, mGluR5 PAMs were developed to bind to the receptor at a site that is distinct from the orthosteric glutamate binding site, and increase the functioning of the receptor in the presence of binding of its endogenous ligand glutamate. The first mGluR5 PAM to be characterized was 3,3'-difluorobenzaldazine (DFB) in 2003 [35], which exhibited poor potency and solubility in aqueous solutions, and was brain impenetrant making it unsuitable for characterization in behavioral assays. A year later, the initial characterization of *N*-[5-chloro-2-[(-1,3-dioxoisoindolin-2-yl)methyl]phenyl]-2-hydroxybenzamide (CPPHA) was described [36], and while this compound exhibited greater potency than DFB, it was also brain impenetrant and thus also not amenable to behavioral studies.

**Figure 1:**
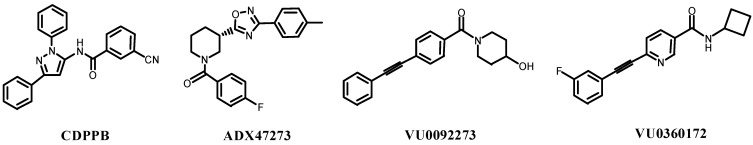
Structure of systemically active mGluR5 PAMs.

A breakthrough in systemically active mGluR5 PAMs that allowed for behavioral assessment of potential antipsychotic efficacy came with the development of 3-cyano-*N*-(1,3-diphenyl-1*H*-pyrazol-5-yl)benzamide (CDPPB) [37,38]. A few years later, the synthesis of (*S*)-(4-fluorophenyl)[3-[3-(4-fluorophenyl)-1,2,4-oxadiazol-5-yl]piperidin-1-yl]methanone (ADX47273) was reported [39] (Figure 1). Both CDPPB and ADX47273 display intrinsic agonist activity at moderate to high concentrations [40,41]. More recently, several other systemically active mGluR5 PAMs have been described, including *N*-methyl-5-(phenylethynyl)pyrimidin-2-amine (MPPA) [42], and(4-hydroxy-piperidin-1-yl)(4-phenylethynyl)phenyl)methanone (VU0092273) [43], the latter of which has been optimized to give the orally active analog *N*-cyclobutyl-6-((3-fluorophenyl)ethynyl)nicotinamide hydrochloride (VU0360172) [43] that has increased selectivity for mGluR5 receptors (Figure 1).

As mentioned earlier, mGluR5 PAMs act on a site of the receptor that is distinct from the orthosteric glutamate binding site. The precise binding site(s) of mGluR5 ligands are frequently assayed by displacement of radiolabeled ligands such as [^3^H]-quisqualate, which binds to the orthosteric glutamate binding site, and [^3^H]3-methoxy-5-(2-pyridinylethynyl)pyridine ([^3^H]-methoxy-PEPy), which binds to an allosteric binding site that is the same as that for the prototypical mGluR5 receptor antagonist 2-methyl-6-(phenylethynyl)pyridine (MPEP) as well as the neutral mGluR5 allosteric modulator 5-methyl-6-(phenylethynyl)-pyridine (5-MPEP) [35,40,44]. With the exception of CPPHA [45], all the aforementioned mGluR5 PAMs appear to bind to the MPEP binding site on the receptor. Unlike CDPPB and ADX47273, the mGluR5 PAMs DFB, CPPHA and MPPA are devoid of any intrinsic agonist activity, and DFB and CPPHA have differential modulatory effects on the activation and phosphorylation of ERK1/2 [46]. As a result of this seemingly different molecular site of action, differing patterns of activation of intracellular signaling cascades, and a relatively shallow structure-activity relationship of CPPHA, recent attempts have been made to utilize the CPPHA chemical scaffold to develop mGluR5 PAMs that do not bind to the MPEP site on the receptor. Such ligands include *N*-(5-chloropyridin-2-yl)-4-propoxybenzamide (VU0001850), 4-butoxy-*N*-(2-fluorophenyl)-benzamide (VU0040237) and 4-butoxy-*N*-(2,4-difluorophenyl)benzamide (VU0357121). These compounds all exhibit high potencies for activating mGluR5 receptors, with EC_50_ concentrations ranging from 33 nM to 1.3 μM [47]. To date, the systemic bioavailability of these compounds as well as their behavioral profiles has not yet been evaluated.

## 4. Antipsychotic and Pro-Cognitive Effects of Systemically Active mGluR5 PAMs

Behavioral studies have shown that CDPPB, ADX47273, MPPA, and VU0360172 have putative antipsychotic-like properties as measured by attenuation of: (1) hyperlocomotion induced by the psychotomimetic compounds ketamine, phencyclidine, and amphetamine [18,37,38,39,43,48], (2) deficits in prepulse inhibition produced by amphetamine [38], and (3) conditioned avoidance responding [39]. In addition, CDPPB has been shown to reverse deficits in cognitive and behavioral flexibility [29,31], negative learning [49], sucrose preference [30], and alterations cortical neuronal activity [50,51,52] produced by the non-competitive NMDA receptor antagonistMK-801. These findings provide evidence for potential antipsychotic efficacy of mGluR5 PAMs, while simultaneously providing additional evidence for glutamatergic mechanisms (i.e., NMDA receptor hypofunction) that underlie some of the symptoms of schizophrenia.

With regards to drug addiction, another neuropsychiatric disorder characterized by deficits in cognition, many studies have shown that pharmacological antagonism of mGluR5 receptors reduces drug reward, reinforcement, and reinstatement of drug-seeking behavior [53,54]. However, recent studies have shown that mGluR5 PAMs may be beneficial in other aspects of drug addiction such as facilitation of the extinction of drug-seeking behavior and reversing drug-induced cognitive deficits. For example, it has been demonstrated that CDPPB facilitates the extinction of a cocaine-associated contextual memory [55] and reduces extinction responding following cocaine self-administration [28,54]. It has also recently been demonstrated that CDPPB reverses deficits in novel object recognition produced by extended access to methamphetamine [56]. Thus, mGluR5 PAMs may be of potential utility as pharmacological adjuncts to cue exposure therapy in the treatment of drug addiction, and may potentially reverse certain cognitive deficits associated with heavy drug use.

Since mGluR5 PAMs indirectly potentiate the function of NMDA receptors, which are critically involved in cellular processes that are believed to underlie learning and memory such as long-term potentiation (LTP) and long-term depression (LTD) of synaptic transmission, one could predict that mGluR5 PAMs might enhance certain forms of synaptic plasticity and learning and memory. Indeed, it has been shown that VU-29 [25] and ADX47273 [18] potentiate LTP and/or LTD in hippocampal slices *in vitro*. Along these lines, behavioral studies have shown that CDPPB and ADX47273 improve the performance of unimpaired mice in the Morris water maze [25], a hippocampus-dependent learning task. Other evidence of potential pro-cognitive effects of mGluR5 PAMs include findings that intracerebroventricular infusion of DFB in rats improved performance in a spatial alternation task [26], while both CDPPB and ADX47273 improved performance in a novel object recognition task [27,39] and the five-choice serial reaction time test [39].

## 5. Summary and Conclusions

While the development of potent, brain penetrant mGluR5 PAMs with favorable selectivity, side effect profiles, and physiochemical properties is still in its relative infancy, preclinical studies thus far suggest that these compounds may represent a novel class of non-monoaminergic antipsychotic medications. In addition, other preclinical studies suggest that mGluR5 PAMs may improve cognitive deficits caused by heavy drug use as well as facilitate the extinction of drug cue reactivity and drug-seeking behavior. Additional studies are needed to determine if mGluR5 PAMs reverse cognitive deficits associated with other neuropsychiatric disorders such as Alzheimer’s disease and other dementias.

Finally, while there is evidence for pro-cognitive effects of mGluR5 PAMs, all such studies to date have been performed in animals in which learning and memory are demonstrated through behavioral changes. Assuming that mGluR5 PAMs will eventually enter clinical trials in human subjects, it remains to be seen whether these compounds have pro-cognitive effects with regards to cognitive functions such as declarative memory, language acquisition, long-term memory recall, *etc*. It also remains to be determined whether the pro-cognitive effects of mGluR5 PAMs are more pronounced in the diseased brain versus that of healthy unimpaired subjects.
